# Labeling of Tannic Acid with Technetium-99m for Diagnosis of Stomach Ulcer

**DOI:** 10.5402/2011/578570

**Published:** 2011-07-19

**Authors:** I. T. Ibrahim, M. El-Tawoosy, H. M. Talaat

**Affiliations:** Radioisotopes Production and Radioactive Sources Division, Labelled Compounds Department, Hot Laboratories Center, Atomic Energy Authority, P.O. Box 13759, Cairo, Egypt

## Abstract

Tannic acid is a polyphenolic compound that could be labeled with technetium-99m. To produce about 90% yield of  ^99m^Tc-tannic acid in acidic media (pH), the conditions required were 150 *μ*g tin chloride, 30 min reaction time, and 200 *μ*g of the substrate. ^99m^Tc-tannic was stable for 6 h. Oral biodistribution of ^99m^Tc-tannic showed that it concentrated in the stomach ulcer to reach about 50% of the total injected dose at 1 h after orall administration. This concentration of ^99m^Tc-tannic in stomach ulcer may be sufficient to radio-image the presence of ulcer in the stomach.

## 1. Introduction

The use of radioisotopes and radiation is indispensable in the research of life science, especially in pharmaceutical sciences. *In vitro* and *in vivo* diagnostic researches in nuclear medicine use radioisotopes and radiopharmaceuticals. Many tracers were used like TC-99m, Re-181, I-131, and others [1–3]. Also many compounds were subjected to be labeled with these radioisotopes like MAG3, MDP, DTPA and others [4–6]. Many items should be in consideration for radio-imaging, for example, radiopharmaceutical development for the kidneys must focus on achieving high-target selectivity and binding affinity, stability and slow metabolism *in vivo*, and minimal nonspecific accumulation and urinary excretion [7].

 A peptic ulcer is defined as mucosal erosions equal to or greater than 0.5 cm of an area of the gastrointestinal tract that is usually acidic and thus extremely painful. As many as 80% of ulcers are associated with *Helicobacter pylori* [8–11], a spiral-shaped bacterium that lives in the acidic environment of the stomach, however only 20% of those cases go to a doctor. Ulcers can also be caused or worsened by drugs such as aspirin and other nonsteroidal anti-inflammatories (NSAIDs) [12]. Contrary to general belief, more peptic ulcers arise in the duodenum (first part of the small intestine, just after the stomach) than in the stomach. About 4% of stomach ulcers are caused by a malignant tumor, so multiple biopsies are needed to exclude cancer [13, 14]. 

 A major causative factor of gastric and of duodenal ulcers is chronic inflammation due to Helicobacter pylori that colonizes the antral mucosa. Thus, the bacterium can cause a chronic active gastritis (type B gastritis), resulting in a defect in the regulation of gastrin production by that part of the stomach, and gastrin secretion can either be decreased (most cases) resulting in hypo- or achlorhydria or increased [15]. Gastrin stimulates the production of gastric acid by parietal cells and, in *H. pylori* colonization responses that increase gastrin [13, 15], the increase in acid can contribute to the erosion of the mucosa and therefore ulcer formation [10, 11]. 

 Another major cause is the use of NSAIDs. The gastric mucosa protects itself from gastric acid with a layer of mucus, the secretion of which is stimulated by certain prostaglandins. NSAIDs block the function of cyclooxygenase 1 (cox-1), which is essential for the production of these prostaglandins [12, 16–19]. 

 Diagnosis of peptic ulcer mainly by An esophagogastroduodenoscopy (EGD), a form of endoscopy, also known as a gastroscopy, is carried out on patients in whom a peptic ulcer is suspected. By direct visual identification, the location and severity of an ulcer can be described. Moreover, if no ulcer is present, EGD can often provide an alternative diagnosis [10, 11]. The diagnosis of Helicobacter pylori can be made by Urea breath test (noninvasive and does not require EGD). So ulcer can be diagnosis by

1. direct culture from an EGD biopsy specimen; this is difficult to do, and can be expensive. Most labs are not set up to perform *H. pylori* cultures,
2. direct detection of urease activity in a biopsy specimen by rapid urease test,
3. measurement of antibody levels in blood (does not require EGD). It is still somewhat controversial whether a positive antibody without EGD is enough to warrant eradication therapy,
4. stool antigen test,
5. histological examination and staining of an EGD biopsy.

Tannic acid (a commercial form of tannin) is a polyphenolic compound [20]. Its weak acidity (pK_a_ around 10) is due to these phenol groups in the structure. Its structure is based mainly on glucose esters of gallic acid. It is a yellow to light brown amorphous powder which is highly soluble in water. Tannic acid is a basic ingredient in the chemical staining of wood. The tannic acid or tannin is already present in woods like oak, walnut, and mahogany. Tannic acid can be applied to woods low in tannin so chemical stains that require tannin content will react. Tannic acid is the most common mordant for cellulose fibers such as cotton. Tannin is often combined with alum and/or iron. The tannin mordant should be done first as metal mordants combine well with the fiber-tannin complex. The presence of tannic acid in the bark of redwood (Sequoia) is a strong natural defense against wildfire, decomposition and infestation. It is found in the seeds, bark, cones, and heartwood [21].

 This study was conducted to label tannic acid with techentium-99m, studying factors affect labeling yield and evaluate the labeled compound with chromatographic methods. It also studied the biodistribution of oral or interavenous ^99m^Tc-tannic acid in normal or mice diseased with stomach ulcer.

## 2. Materials and Methods

### 2.1. Drugs and Chemicals

1. Tchentium-99m was obtained as saline eluent of an expired Mo column.
2. Tannic acid was supplied from ICN Chemical Company, USA.
3. Tin chloride was purchased from Sigma Chemical Company, USA.
4. All other chemical reagents were of analytical grade (AR), obtained from reputed manufacturers.

### 2.2. Animals

Female Swiss Albino mice weighing 20–25 gm were purchased from the Institute of Eye Research Cairo, Egypt. The animals were kept at constant environmental and nutritional conditions throughout the experimental period and kept at room temperature (22 ± 2)°C with a 12 hr on/off light schedule. Animals were kept with free access to food and water all over the experiment.

### 2.3. Methods

#### 2.3.1. Labeling Procedure and Requirement

^99m^Tc-tannic acid was prepared by the following procedures [22]. 1 mg tannic acid was dissolved in 1 mL purged distilled water with stirring. Tin chloride was added to tannic acid solution in evacuated vial with Hamilton syringe and approximately 200–400 MBq ^99m^Tc at room temperature. After a specified interval of time, chromatographic analysis was developed using paper chromatography ascending techniques [23]. The yield of the reaction and the radiochemical purity were determined by paper chromatography using acetone as mobile phase to distinguish between free pertechnetate at the top and both complex and reduced colloids near the point of spotting. On the other hand, Thin Layer Chromatography using 2N HCl as a mobile phase differentiates between reduced colloids which persist near the point of spotting and both complex and free, which move towards the front of chromatogram.

### 2.4. Factors Affecting % Labeling Yield

This experiment was conducted to study the different factors that affect labeling yield such as (1) tin content, (2) substrate content, (3) pH of the reaction, and (4) reaction time. 

In the process of labeling, trials and errors were performed for each factor under investigations till obtains the optimum value. The experiment was repeated with all factors kept at optimum changing except the factor under study, till the optimal conditions are achieved [24].

### 2.5. In Vitro Stability

This experiment was conducted to determine the stability of ^99m^Tc-tannic acid after labeling and the impact of time on that compound. The yield was measured at different time intervals (1, 2, 6, 12 and 24 h) after labeling [25].

### 2.6. Induction of Ulcer in the Stomach of Mice

There are many methods for induction of ulcer in laboratory animals. The simplest one in mice is the oral administration of ethyl alcohol 1 mL/mice. After 1-2 h the stomach showed distension of stomach and symptoms of ulcer appeared [26].

### 2.7. In Vivo Biodistribution

#### 2.7.1. In Normal Mice

*In vivo* biodistribution studies were performed using 3 groups each comprise six mice. Each animal was injected in the tail vein with 0.2 mL solution containing 200–400 KBq of ^99m^Tc-tannic acid freshly prepared. The mice were kept in metabolic cages for the required time. Each group was subjected to scarification by cervical dislocation at the recommended time (15 min, 1 hr or 2 hr) after injection. Organs or tissues of interest were removed, washed with saline, weighted, and counted. Correction was made for background radiation and physical decay during the experiment. The weights of blood, bone, and muscles were assumed to be 7, 10, and 40% of the total body weight, respectively, [27].

#### 2.7.2. In Ulcer-Bearing Mice

Biodistribution of ^99m^Tc-tannic acid was carried out in two groups of animals each group consists of 18 ulcer-bearing mice. The first group received 200–400 KBq of ^99m^Tc-tannic/mice intravenously in the tail vein and the other group received oral 200–400 KBq of ^99m^Tc-tannic/mice using oral tube. Each group subdivided to 3 subgroups of 6 mice each. Animals in each group were kept in metabolic cages for scarification at its required time, after 15 min, 1 h or 2 h after injection of the labeled drug. Sacrification of mice was done by cervical dislocation and the organs or tissues of interest were isolated, weighted and counted for its uptake of radioactivity. The counting tubes, including a standard equivalent to 1% of the injected dose, were assayed in a well type NaI (TI) gamma counter and the results were calculated as percentages of injected dose (ID) per gram tissue. The final results were expressed as mean ± one standard error [28].

### 2.8. Statistical Analysis

The results are expressed as means ± SEM for the indicated number of different experiments. The statistical significance of differences was assessed by unpaired Student's *t*-test *P* < 0.05.

## 3. Results and Discussion

### 3.1. Paper Chromatography

The analysis of chromatographic data revealed the high percentage labeling yield of ^99m^Tc-tannic acid. Free pertechnetate was obtained from paper acetone chromatogram. Colloid was obtained from 2N HCl chromatogram. Complex ^99m^Tc-tannic acid was obtained by subtracting % colloid from % activity obtained near the spotting in acetone chromatogram.

### 3.2. Factors Affecting Labeling Yield

#### 3.2.1. Tin Content

**Figure 1:**
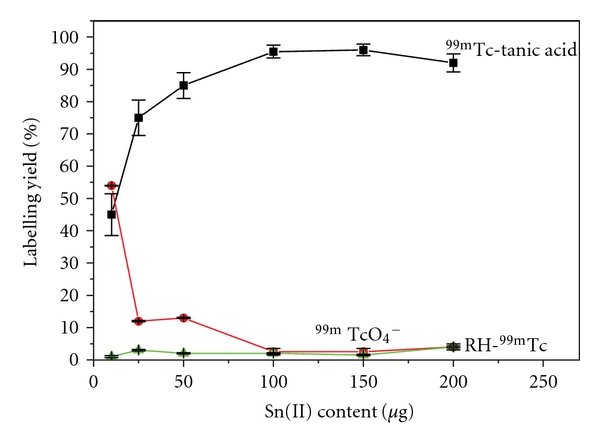
Effect of tin chloride content on the radiochemical yield ^99m^Tc-tannic acid.

Results obtained in this study showed the high yield obtained for ^99m^Tc-tannic acid using tin chloride as reducing agent (Figure 1). It was observed that the radiochemical yield significantly increased by increasing the amount of tin from 10 *μ*g to 150 *μ*g (optimum content) at which maximum labeling yield was obtained. By increasing the amount of tin to 200 *μ*g, the yield showed significant decrease in % complex ^99m^Tc-tannic acid. A significant reduction in the labeling yield was noted by decreasing the concentration of tin below 100 *μ*g may be explained as at low concentrations of tin, not all ^99m^Tc was reduced. While by increase the tin content to 200 *μ*g colloid may be increased and hence, % labeled complex decreased [29].

#### 3.2.2. Effect of Substrate Content

**Figure 2:**
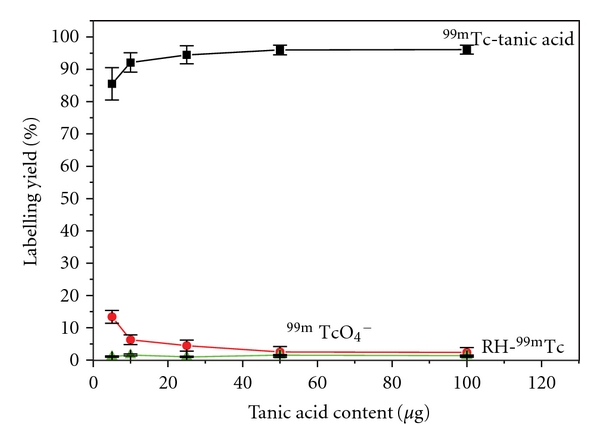
Effect of tannic acid content on the labeling yield.

The influence of tannic acid content as a substrate on the labeling yield using tin chloride was shown in Figure 2. The increase of the concentration of tannic acid was accompanied by a significant increase in the labeling yield, where it reached above 95% at 50 *μ*g of tannic acid. Increasing the amount of tannic acid above 50 *μ*g does not affecting the labeling yield. Increasing the concentration of starting material is usually increases the total incorporation of ^99m^Tc-tannic acid since there is a minimum limit to the volume used [30]. 50 *μ*g of tannic acid was required to obtain maximum labeling yield, below this concentration significant decrease in the yield.

#### 3.2.3. Effect of pH

**Figure 3:**
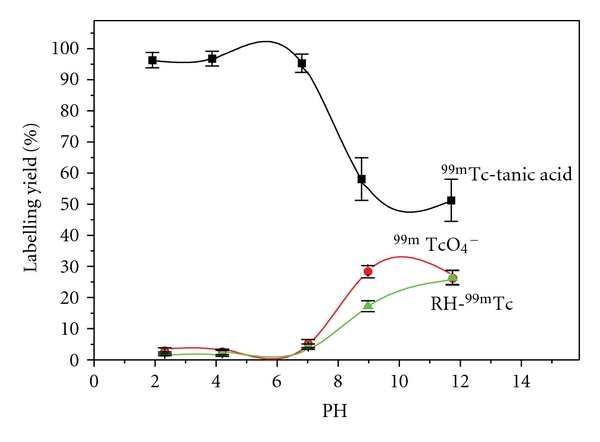
Effect of pH of the reaction medium on the labeling yield of  ^99m^Tc-tannic acid.

In order to reach the suitable pH value for maximum radiochemical yield, labeling of tannic acid with ^99m^Tc was carried out at different pH values ranging from 2–12. The test was performed using 50 *μ*g of tannic acid, 100 *μ*L of 0.5 M phosphate buffer of pH7 at 30 min reaction time. The experiment was repeated using 100 *μ*L of each buffer at different pH values. As shown in Figure 3, pH 7 is the optimum pH at which the maximum yield was obtained (95.5%). Also, it was observed that at pH 2 or 4, the yield was 95%, while at pH values 9 and 12; the yield was 57%, 50%, respectively. There was significant difference between all pH values of the reaction mediums.

#### 3.2.4. Effect of Reaction Time

**Figure 4:**
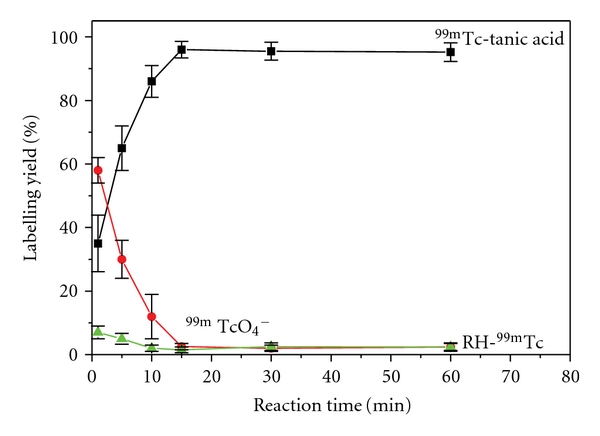
Effect of reaction time on the % labeling yield of  ^99m^Tc-tannic acid.

Figure 4 shows the relationship between the reaction time and the yield of  ^99m^Tc-tannic acid. Radiochemical yield was significantly increased from 35% to 95.5% with increasing reaction time from 1 min to 30 min. Extending the reaction time more than 30 min does not affect the radiochemical yield. The efficiency of reducing agent may be affected by time and thus yield decreased [31].

### 3.3. In Vitro Stability of ^99m^Tc-Tannic Acid

In the present experiment, a very slight decrease in the stability of ^99m^Tc-tannic acid from 95.5% to 94.5% at 24 h after labeling was observed. Approximately, a constant labeling yield was observed at all time intervals after labeling, as the yield was 95.5, 95.1, 95.2, 94.7 and 94.5% at 1 h, 2 h, 6 h, 12 h and 24 h, respectively, as shown in Table 1. 

### 3.4. Biodistribution of  ^99m^Tc-Tannic Acid

#### 3.4.1. In Normal Mice

Biodistribution study of  ^99m^Tc-tannic acid in normal mice showed that ^99m^Tc-tannic acid was distributed rapidly in blood, liver, lung and intestine at 15 min after injection. After 1h, ^99m^Tc-tannic acid uptake was significantly decreased in organs like blood, heart, liver and intestine. However, ^99m^Tc-tannic acid uptake was significantly increased in stomach after 1 h. At 2 h after injection, the majority of tissues showed significant decrease in ^99m^Tc-tannic acid uptake as shown in Table 2. 

#### 3.4.2. In Ulcer Bearing Mice

**(1) Intravenous Route**

The results of this experiment showed that the sites of greatest uptake of ^99m^Tc-tannic acid after 15 minutes after injection were the blood and stomach (18 and 14.2), respectively.  Table 3 shows that the concentration of ^99m^Tc-tannic acid was the lowest in muscle, kidney and spleen at 15 minutes after injection. No significant change in the uptake of ^99m^Tc-tannic acid at 2 h after injection was observed when compared to its previous value. The data also showed that some organs exhibit significant increase of uptake at 1 h after injection like kidney and muscle. On the other hand, significant decrease in ^99m^Tc-tannic acid uptake was observed in blood, heart, spleen and lung at the same time. At 2 h after injection, the majority of organs showed significant decrease in uptake of ^99m^Tc-tannic acid.

**(2) Oral Administration in Ulcer Bearing Mice**

After oral administration of ^99m^Tc-tannic acid in ulcer-bearing mice, it was found that ^99m^Tc-tannic acid uptake was the greatest in blood, liver and stomach (8.5, 3.5 and 18, resp.) at 15 minutes after injection and lowest in heart, spleen, lung and kidney (1, 1, 2.1 and 1, resp.) (Table 4). The uptake of ^99m^Tc-tannic acid in stomach was significantly increased with time at 1 h after injection, as it was 50% per g, then, it was significantly decreased to 20% at 2 h after injection.

## 4. Conclusion

Incorporation of ^99m^Tc-tannic acid to an ulcer was achieved by oral administration of ^99m^Tc-tannic acid in ulcer-bearing mice. The appropriate conditions for labeling (95.5% yield) were 100 *μ*g tannic acid, 50 *μ*g tin as reducing agent, at pH 7, at room temperature and 30 minutes reaction time. The great incorporation of ^99m^Tc-tannic acid in ulcer sites facilitates stomach imaging. ^99m^Tc-tannic acid was found to be highly localized in ulcer sites specially after oral adminstration. In conclusion, this study demonstrates a hopeful approach for stomach ulcer imaging.

## Figures and Tables

**Table 1:** Effect of time on the stability of ^99m^Tc-tannic acid.

Time after labeling (h)	% Labeled compound	% Free **^99m^**Tc	% Colloid
1 h	95.5 ± 0.52	2.5 ± 0.05	2.0 ± 0.05
2 h	95.1 ± 0.25	2.7 ± 0.25	2.2 ± 0.05
6 h	95.2 ± 0.25	2.8 ± 0.25	2.0 ± 0.45
12 h	94.7 ± 0.50	2.9 ± 0.25	2.1 ± 0.50
24 h	94.5 ± 0.30	2.1 ± 0.15	3.2 ± 0.35

Values represent the mean ± SEM *n* = 6.

**Table 2:** Biodistribution of ^99m^Tc-tannic acid in normal mice.

Organs and Body fluids	Percent I.D./gram organ		
	Time after injection		
	15 min	1 hr	2 hr
Blood	18.5 ± 1.10	11.2 ± 0.20*	7.2 ± 0.04*
Bone	4.1 ± 0.05	3.6 ± 0.10*	1.4 ± 0.10*
Muscle	3.0 ± 0.01	2.9 ± 0.02*	1.9 ± 0.10
Liver	8.1 ± 0.05	7.7 ± 0.15*	5.4 ± 0.16*
Lung	6.0 ± 0.10	4.5 ± 0.12*	2.3 ± 0.20*
Heart	5.1 ± 0.50	3.6 ± 0.50*	1.3 ± 0.01*
Stomach	5.0 ± 0.90	6.5 ± 0.60	3.4 ± 0.16*
Intestine	7.0 ± 0.50	5.4 ± 0.30*	4.1 ± 0.10*
Kidney	2.7 ± 0.40	4.6 ± 0.60	4.2 ± 0.30*
Spleen	0.5 ± 0.02	0.9 ± 0.24*	0.8 ± 0.2

Values represent mean ± SEM *n* = 10

*significantly different from previous value of each organ using unpaired Student's *t*-test (*P* < 0.05).

**Table 3:** Intravenous biodistribution of ^99m^Tc-tannic acid in ulcer-bearing mice.

Organs and body fluids	Percent I.D./gram organ		
	Time after injection		
	15 min	1 hr	2 hr
Blood	18.0 ± 1.10	10.1 ± 0.20*	8.0 ± 0.04*
Bone	6.0 ± 0.05	3.4 ± 0.10*	3.5 ± 0.10*
Muscle	2.9 ± 0.01	4.0 ± 0.02*	7.2 ± 0.10
Liver	9.0 ± 0.05	2.0 ± 0.15*	1.1 ± 0.16*
Lung	2.2 ± 0.10	1.3 ± 0.12*	0.1 ± 0.20*
Heart	4.7 ± 0.50	2.7 ± 0.50*	1.3 ± 0.01*
Stomach	14.2 ± 0.90	7.0 ± 0.60	15.0 ± 0.16*
Intestine	7.3 ± 0.50	7.5 ± 0.30	6.0 ± 0.10*
Kidney	5.0 ± 0.40	7.0 ± 0.60	4.5 ± 0.30*
Spleen	3.0 ± 0.02	1.2 ± 0.24*	1.1 ± 0.2

Values represent mean ± SEM *n* = 6

*Significantly different from previous value of each organ using unpaired Student's *t*-test (*P* < 0.05).

**Table 4:** Oral administration of ^99m^Tc-tannic acid in ulcer-bearing mice.

Organs andbody fluids	Percent I.D./gram organ		
	Time after injection		
	15 min	1 hr	2 hr
Blood	8.5 ± 1.10	7.0 ± 0.20*	3.5 ± 0.04*
Bone	4.0 ± 0.05	3.0 ± 0.10*	2.0 ± 0.10*
Muscle	3.0 ± 0.01	2.0 ± 0.02*	2.2 ± 0.10
Liver	3.5 ± 0.05	2.1 ± 0.15*	3.2 ± 0.16*
Lung	2.1 ± 0.10	3.1 ± 0.12*	3.5 ± 0.20*
Heart	1.0 ± 0.50	4.0 ± 0.50*	5.0 ± 0.01*
Stomach	18.0 ± 0.90	50.0 ± 0.60	20.0 ± 0.16*
Intestine	4.0 ± 0.50	14.0 ± 0.30*	7.0 ± 0.10*
Kidney	1.0 ± 0.40	2.5 ± 0.60	3.5 ± 0.30*
Spleen	1.0 ± 0.02	1.5 ± 0.24*	2.5 ± 0.20

Values represent mean ± SEM *n* = 6

*Significantly different from previous value of each organ using unpaired Student's *t*-test (*P* < 0.05).
